# Association between Age and Changes in Heart Rate Variability after Hemodialysis in Patients with Diabetes

**DOI:** 10.3389/fnagi.2018.00043

**Published:** 2018-02-20

**Authors:** Sheng-Wen Niu, Jiun-Chi Huang, Szu-Chia Chen, Hugo Y.-H. Lin, I.-Ching Kuo, Pei-Yu Wu, Yi-Wen Chiu, Jer-Ming Chang

**Affiliations:** ^1^Division of Nephrology, Department of Internal Medicine, Kaohsiung Medical University Hospital, Kaohsiung Medical University, Kaohsiung, Taiwan; ^2^Department of Internal Medicine, Kaohsiung Municipal Ta-Tung Hospital, Kaohsiung Medical University Hospital, Kaohsiung Medical University, Kaohsiung, Taiwan; ^3^Graduate Institute of Clinical Medicine, College of Medicine, Kaohsiung Medical University, Kaohsiung, Taiwan; ^4^Department of Internal Medicine, Kaohsiung Municipal Hsiao-Kang Hospital, Kaohsiung Medical University, Kaohsiung, Taiwan; ^5^Faculty of Medicine, College of Medicine, Kaohsiung Medical University, Kaohsiung, Taiwan; ^6^Graduate Institute of Medicine, College of Medicine, Kaohsiung Medical University, Kaohsiung, Taiwan; ^7^Department of Internal Medicine, Kaohsiung Municipal Cijin Hospital, Kaohsiung Medical University, Kaohsiung, Taiwan

**Keywords:** heart rate variability, autonomic nervous system, age, diabetes, hemodialysis

## Abstract

**Background:** Heart rate variability (HRV) represents changes in the time between successive heart beats, and it has been used to assess the autonomic nervous system. Previous studies have reported autonomic dysfunction in diabetic patients undergoing hemodialysis (HD), however, no studies have evaluated the effects of age on changes in HRV in these patients. The aim of this study was to examine the effects of age on changes in HRV in diabetic HD patients.

**Methods:** We enrolled 84 diabetic patients receiving maintenance HD. HRV was measured before and after HD to assess changes in HRV (ΔHRV). The patients were divided into two groups based on their age (65 years< or ≥65 years).

**Results:** Compared to the patients aged <65 years, those aged ≥65 years had a higher high frequency (HF) % (*p* = 0.032) before HD. The patients aged <65 years had a significant increase in very low frequency, low frequency (LF), and HF after HD. The patients aged ≥65 years had a significant increase in LF, but a significant decrease in HF% after HD. There was a significant interaction between age and change of HF% (*p* = 0.023) after HD. After multivariate adjustments for clinical, biochemical data and medications, systolic blood pressure, total cholesterol, hemoglobin, and hemoglobin were associated with ΔLF, whereas cerebrovascular disease, systolic blood pressure, and fasting glucose were associated with ΔHF% in patients aged ≥65 years.

**Conclusion:** Our study demonstrated significant changes in HRV after HD in diabetic patients. In the patients aged ≥65 years, LF was increased, whereas HF% was decreased significantly after HD. Among the HRV parameters, age had an interaction with the change of HF%.

## Introduction

Heart rate variability (HRV) is a non-invasive method of measuring variations in heart rate, and it can be used to evaluate cardiovascular (CV) autonomic neuropathy and activities of the autonomic nervous system (Camm et al., 1996). In practice, HRV is defined as variations in instantaneous heart rate and R–R intervals measured using electrocardiography (ECG) (Camm et al., 1996). Abnormal HRV primarily reflects dysregulation between the sympathetic and parasympathetic nervous systems. Frequency-domain analysis of HRV has been increasingly used to assess the autonomic nervous system due to its non-invasiveness and availability.

A low HRV, indicating impaired autonomic function, has been reported in patients undergoing hemodialysis (HD), and it has been associated with adverse CV outcomes and even death in patients undergoing HD (Fukuta et al., 2003; Oikawa et al., 2009). Our recent study demonstrated that changes in HRV parameters before and after HD have a stronger predictive ability for overall and CV mortality than HRV parameters before HD (Chen et al., 2016). A previous study reported that HD patients with diabetes had a smaller degree of changes in HRV after HD than patients without diabetes, and that this may be related to diabetic autonomic neuropathy (Zitt et al., 2008). However, the factors associated with changes in HRV in diabetic HD patients have yet to be clarified.

Many neurological diseases of in the elderly such as dementia (Galimberti and Scarpini, 2016), Parkinson’s disease (Pelosin et al., 2016) and cognitive impairment (Liu et al., 2017) have been reported to be caused by age-related cholinergic dysfunction. In addition to neurological diseases, cholinergic degeneration has also been reported to play a role in an obstructed bladder (Silva-Ramos et al., 2015) and constipation in the elderly, and especially in those with diabetes (Bharucha et al., 2013). The number of elderly patients undergoing HD has increased along with life expectancy, and these patients have been reported to be frailer (Fried et al., 2001) with greater functional and cognitive impairments compared to those who do not require HD (Anand et al., 2010). However, little is known about the association between age and autonomic regulation in HD patients with diabetes mellitus (DM). Therefore, the aims of this study were to investigate the relationship between age and HRV parameters before and after HD, and further investigate the factors associated with change in HRV in diabetic patients.

## Materials and Methods

### Study Patients and Design

This study was conducted at a regional hospital in southern Taiwan, and included all maintenance HD patients except for those receiving HD at night. A total of 84 diabetic patients (45 males and 39 females) were enrolled from May 2012 to July 2012. All of these patients underwent HD three times per week, with each session lasting for 3.5–4.5 h, a blood flow rate of 250–300 mL/min, and a dialysate flow of 500 mL/min. *K*t/*V* was calculated from blood samples obtained before and after HD.

### Ethics Statement

The study protocol was approved by the Institutional Review Board of Kaohsiung Medical University Hospital. Written informed consent was obtained from all of the enrolled patients, and the study was conducted according to the principles expressed in the Declaration of Helsinki. In addition, all of the patients consented to the inclusion and publication of their data in this study.

### Electrocardiogram Signal Processing

Short-term power spectral analysis of HRV was performed in all of the enrolled patients in a quiet room with a temperature of 28°C. The HRV analysis was performed according to standard protocols as reported previously (Kuo et al., 1999; Liu et al., 2003; Chen et al., 2009). Pericardial ECG was performed continuously for 5 min with the patients lying quietly in the supine position and breathing normally for at least 10 min. The ECG examinations were performed 30 min before and 30 min after HD during the day (8 a.m. to 5 p.m.), and the signals were recorded using an HRV analyzer (SS1C, Enjoy Research, Taipei, Taiwan) which was equipped with an analog-to-digital converter and a sampling rate of 256 Hz. The digitized ECG signals were acquired, stored, and processed using a computer. During analysis, each QRS complex was identified, and premature ventricular complexes and noise were removed using a standard QRS template using the software provided by the HRV analyzer. This was followed by resampling of the stationary *R*–*R* values with interpolation at 7.11 Hz to provide consistency over a time period (Kuo et al., 1999).

### HRV Frequency-Domain Analysis

Frequency-domain analysis was conducted using fast Fourier transformation. The direct current component was first removed, and then the effect of leakage was reduced using a Hamming window (Kuo and Chan, 1993). The power spectrum density was estimated based on FFT for each time segment (288 s; 2048 data points), and attenuation of the resulting power spectrum caused by sampling and the Hamming window was corrected. The power spectrum was then quantified into standard frequency-domain measurement including very low frequency (VLF) (0.003–0.04 Hz), high frequency (HF) (0.15–0.40 Hz), low frequency (LF) (0.04–0.15 Hz), total power (0.003–0.40 Hz) and LF/HF ratio HRV as previously reported (Camm et al., 1996). HF was normalized to the percentage of total power to detect the sympathetic influence on HRV as follows: HF% = HF/(total power-VLF)^∗^100%. All of the HRV parameters were logarithmically transformed to correct for skewness of the distribution (Camm et al., 1996).

### Collection of Demographic, Medical, and Laboratory Data

Demographic and medical data were obtained from medical records and patient interviews, and included age, sex and co-morbidities. An autoanalyzer (COBAS Integra 400, Roche Diagnostics GmbH, Mannheim, Germany) was used for all laboratory examinations which were conducted using fasting blood samples. The serum concentration of intact parathyroid hormone (iPTH) was measured using a commercially available two-sided immunoradiometric assay (CIS Bio International, Saclay, France). Ultrafiltration rate was defined as ultrafiltration divided by body weight. *K*t/*V* was used as a measure of dialysis efficiency, and was determined monthly using the Daugirdas method (Daugirdas, 1993).

### Statistical Analysis

Statistical analysis was performed using SPSS for Windows version 15.0 (SPSS Inc. Chicago, IL, United States). HRV parameters are presented as percentage, mean ± standard deviation, and mean ± standard error of the mean. The duration of dialysis and levels of serum triglycerides and iPTH are presented as median (25th–75th percentile). Differences between groups were analyzed using the chi-square test for categorical variables and the independent *t*-test for continuous variables with an approximately normal distribution, or the Mann–Whitney *U* test for continuous variables with a skewed distribution. The Wilcoxon signed-rank test using non-parametric statistical analysis was carried out for comparisons of the HRV parameters before and after HD. Furthermore, two-ways repeated measures analysis of variance was used to test the interaction between age and change of HRV parameters after HD. Changes in the HRV (ΔHRV) parameters were calculated as those measured after HD minus those measured before HD. Multiple stepwise linear regression analysis was used to identify the factors associated with ΔHRV parameters. A *p*-value of less than 0.05 was considered to indicate a significant difference.

## Results

The mean age of the 84 enrolled patients with DM undergoing maintenance HD was 62.6 ± 10.1 years. Of these patients, 53.6% patients were male and 41.7% were ≥65 years old. Comparisons of the baseline characteristics between the patients aged <65 years and ≥65 years are shown in **Table 1**. Compared to the patients younger than 65 years, those ≥65 years of age were more predominantly female, and had lower systolic and diastolic blood pressures, lower total cholesterol, lower low-density lipoprotein (LDL) cholesterol and lower creatinine. Among all of the pre-dialysis HRV parameters, only HF% was significantly higher in the patients ≥65 years of age compared to those <65 years.

**Table 1:** Comparison of baseline characteristics between diabetes mellitus (DM) patients with age <65 and ≥65 years old.

Characteristics	All patients (*N* = 84)	Age <65 years (*N* = 49)	Age ≥65 years (*N* = 35)	*p*
Age (year)	62.6 ± 10.1	56.2 ± 7.1	71.5 ± 6.1	<0.001
Male gender (%)	53.6	63.3	40.0	0.035
Duration of dialysis (year)	3.3 (1.3–6.7)	2.9 (1.0–5.3)	5.2 (1.7–9.1)	0.082
Smoking history (%)	27.4	32.7	20.0	0.200
Hypertension (%)	77.4	81.6	71.4	0.270
Coronary artery disease (%)	38.1	34.7	42.9	0.448
Cerebrovascular disease (%)	31.0	28.6	34.3	0.576
Systolic blood pressure (mmHg)	164.0 ± 26.0	169.6 ± 24.5	155.6 ± 26.2	0.017
Diastolic blood pressure (mmHg)	84.1 ± 14.8	87.8 ± 13.9	78.6 ± 14.6	0.006
Laboratory parameters				
Albumin (g/dL)	3.8 ± 0.3	3.8 ± 0.3	3.7 ± 0.3	0.051
Fasting glucose (mg/dL)	144.0 ± 62.8	133.4 ± 47.2	158.0 ± 77.3	0.102
Triglycerides (mg/dL)	152.5 (104–228.5)	177 (104.8–240.8)	132.5 (94.5–177.8)	0.156
Total cholesterol (mg/dL)	175.4 ± 44.2	188.1 ± 40.7	157.5 ± 43.2	0.002
Hemoglobin (g/dL)	10.3 ± 1.2	10.5 ± 1.0	10.1 ± 1.5	0.143
Creatinine (mg/dL)	9.3 ± 1.9	9.8 ± 1.6	8.5 ± 2.0	0.002
Potassium (mEq/L)	4.5 ± 0.7	4.5 ± 0.6	4.4 ± 0.7	0.499
Total calcium (mg/dL)	9.2 ± 1.0	9.3 ± 0.9	9.1 ± 1.0	0.439
Phosphorous (mg/dL)	4.6 ± 1.1	4.6 ± 1.1	4.5 ± 1.1	0.634
Calcium-phosphorous product (mg^2^/dL^2^)	41.8 ± 11.5	42.8 ± 11.6	40.4 ± 11.4	0.359
iPTH (pg/mL)	327.8 (183.8–463.3)	359.8 (193.0–498.7)	306.5 (177.9–364.9)	0.525
Uric acid (mg/dL)	7.6 ± 1.9	7.8 ± 1.8	7.3 ± 2.0	0.227
Kt/V (Daugirdes)	1.5 ± 0.3	1.5 ± 0.2	1.6 ± 0.4	0.653
Ultrafiltration rate (%)	4.4 ± 1.4	4.3 ± 1.3	4.6 ± 1.6	0.415
Pre-dialysis HRV parameters				
VLF (ln ms^2^)	3.59 ± 0.20	3.38 ± 0.21	3.89 ± 0.38	0.215
LF (ln ms^2^)	1.39 ± 0.48	0.73 ± 0.77	2.32 ± 0.38	0.104
HF (ln ms^2^)	1.60 ± 0.54	0.80 ± 0.88	2.72 ± 0.38	0.082
HF% (nu)	35.5 ± 1.7	32.5 ± 2.1	39.8 ± 2.6	0.032
LF/HF ratio	–0.21 ± 0.14	–0.07 ± 0.19	–0.40 ± 0.19	0.243
Post-dialysis HRV parameters				
VLF (ln ms^2^)	4.19 ± 0.19	4.01 ± 0.24	4.45 ± 0.29	0.259
LF (ln ms^2^)	2.28 ± 0.50	2.02 ± 0.61	2.64 ± 0.86	0.553
HF (ln ms^2^)	2.05 ± 0.55	1.86 ± 0.67	2.31 ± 0.95	0.696
HF% (nu)	29.6 ± 1.4	30.4 ± 1.8	28.4 ± 2.4	0.516
LF/HF ratio	0.23 ± 0.13	0.16 ± 0.16	0.34 ± 0.22	0.508
Medications				
ACEI and/or ARB use	28.6	28.2	29.2	0.935
Beta-blocker use	30.2	25.6	37.5	0.319
Calcium channel blocker use	31.7	28.2	37.5	0.441

*LDL, low-density lipoprotein; iPTH, intact parathyroid hormone; HRV, heart rate variability; LF, low frequency; HF, high frequency; ACEI, angiotensin converting enzyme inhibitor; ARB, angiotensin II receptor blocker.*

### Changes in HRV Parameters before and after HD

In the patients aged <65 years, VLF (*p* = 0.014), LF (*p* < 0.001), and HF (*p* = 0.030), were significantly increased after HD. In comparison, LF (*p* = 0.043) was significantly increased in the patients ≥65 years of age, but HF% (*p* < 0.007) was significantly decreased after HD (**Table 2**). Two-ways repeated measures analysis of variance was carried out to elucidate the interaction between age and change of HRV parameters after HD. Of note, the interaction between age and change of HF% was significant (*p* = 0.023). There was no significant interaction between age and change of VLF (*p* = 0.852), LF (*p* = 0.316), HF (*p* = 0.189), and LF/HF ratio (*p* = 0.117).

**Table 2:** Heart rate variability (HRV) parameters of DM patients with age <65 and ≥65 years old before and after hemodialysis.

HRV parameters (frequency domain)	Age <65 years			Age ≥65 years		
	Before hemodialysis	After hemodialysis	*p*	Before hemodialysis	After hemodialysis	*p*
VLF (ln ms^2^)	3.75 (2.78–4.23)	4.03 (2.96–5.43)	0.014	4.21 (2.89–4.77)	4.34 (3.33–5.54)	0.176
LF (ln ms^2^)	1.52 (0.69–2.44)	2.34 (1.41–3.81)	<0.001	2.55 (1.36–3.26)	3.43 (1.53–4.63)	0.043
HF (ln ms^2^)	1.89 (0.94–2.78)	2.31 (1.50–3.11)	0.030	2.98 (1.88–3.85)	2.53 (1.74–3.90)	0.905
HF% (nu)	31.2 (20.1–43.1)	29.8 (22.2–37.8)	0.885	39.5 (29.4–51.0)	28.5 (18.1–39.0)	0.007
LF/HF ratio	–0.19 (–1.11–0.77)	0.11 (–0.36–0.91)	0.665	–0.14 (–0.98–0.48)	0.25 (–0.52–1.17)	0.091

*LDL, low-density lipoprotein; iPTH, intact parathyroid hormone; HRV, heart rate variability; LF, low frequency; HF, high frequency; ACEI, angiotensin converting enzyme inhibitor; ARB, angiotensin II receptor blocker.*

### Determinants of ΔHRV Parameters in the Patients <65 Years Old

**Table 3** shows the unstandardized β coefficients of ΔVLF, ΔLF, and ΔHF which were significant in **Table 2** in the patients <65 years old, after adjusting for age, sex, duration of HD, a history of smoking, hypertension, coronary artery disease and cerebrovascular disease, systolic and diastolic blood pressure, albumin, fasting glucose, triglycerides, total cholesterol, LDL-cholesterol, hemoglobin, creatinine, potassium, total calcium, phosphorous, calcium-phosphorous product, iPTH, uric acid, *K*t/*V*, ultrafiltration rate and medication. In the multivariate stepwise analysis, cerebrovascular disease (β = -1.160, *p* = 0.015), diastolic blood pressure (β = -0.018, *p* = 0.017), fasting glucose (β = -0.016, *p* = 0.001), hemoglobin (β = -0.897, *p* = 0.001), and beta-blocker use (β = 1.103, *p* = 0.009) were independently associated with ΔVLF. Diastolic blood pressure (β = -0.029, *p* = 0.030), and ultrafiltration rate (β = -0.272, *p* = 0.033) were associated with ΔLF. Cerebrovascular disease (β = -0.765, *p* = 0.049) was associated with ΔHF.

**Table 3:** Determinants of ΔHRV parameters of DM patients with age <65 years old.

ΔHRV parameters	Multivariate (stepwise)	
	Unstandardized coefficient β (95% CI)	*p*
ΔVLF		
Cerebrovascular disease	–1.160 (-2.074, –0.245)	0.015
Diastolic blood pressure (per 1 mmHg)	–0.018 (–0.032, –0.003)	0.017
Fasting glucose (per 1 mg/dL)	–0.016 (–0.025, –0.007)	0.001
Hemoglobin (per 1 g/dL)	–0.897 (–1.407, –0.387)	0.001
Beta-blocker use	1.103 (0.299, 1.906)	0.009
ΔLF		
Diastolic blood pressure (per 1 mmHg)	–0.029 (–0.003, –0.054)	0.030
Ultrafiltration rate (per 1 %)	–0.272 (–0.024, –0.520)	0.033
ΔHF		
Cerebrovascular disease	–0.765 (–1.527, –0.003)	0.049

*Values expressed as unstandardized coefficient β and 95% confidence interval (CI). Covariates in the multivariate model included age, sex, duration of dialysis, a history of smoking, hypertension, coronary artery disease and cerebrovascular disease, systolic and diastolic blood pressure, albumin, fasting glucose, triglycerides, total cholesterol, LDL-cholesterol, hemoglobin, creatinine, potassium, total calcium, phosphorous, calcium-phosphorous product, iPTH, uric acid, Kt/V, ultrafiltration rate, and medications use including ACEI and/or ARB, Beta-blocker and calcium channel blocker.*

### Determinants of ΔHRV Parameters in the Patients ≥65 Years Old

**Table 4** presents the unstandardized β coefficients of ΔLF and ΔHF% which were significant in **Table 2** in the patients ≥65 years old after adjusting for demographic, clinical and biochemical characteristics and medications. In the multivariate stepwise analysis, systolic blood pressure (β = 0.060, *p* = 0.023), total cholesterol (β = -0.146, *p* < 0.001) and hemoglobin (β = 1.466, *p* < 0.001) were associated with ΔLF. Furthermore, cerebrovascular disease (β = 12.762, *p* = 0.047), systolic blood pressure (β = 0.357, *p* = 0.012), and fasting glucose (β = -0.126, *p* = 0.003) were associated with ΔHF%.

**Table 4:** Determinants of ΔHRV parameters of DM patients with age ≥65 years old.

ΔHRV parameters	Multivariate (stepwise)	
	Unstandardized coefficient β (95% CI)	*p*
ΔLF		
Systolic blood pressure (per 1 mmHg)	0.060 (0.009, 0.111)	0.023
Total cholesterol (per 1 mg/dL)	–0.146 (–0.173, –1.119)	<0.001
Hemoglobin (per 1 g/dL)	1.466 (0.822, 2.109)	<0.001
ΔHF %		
Cerebrovascular disease	12.762 (0.216, 25.309)	0.047
Systolic blood pressure (per 1 mmHg)	0.357 (0.090, 0.625)	0.012
Fasting glucose (per 1 mg/dL)	–0.126 (–0.204, –0.048)	0.003

*Covariates in the multivariate model included age, sex, duration of dialysis, a history of smoking, hypertension, coronary artery disease and cerebrovascular disease, systolic and diastolic blood pressure, albumin, fasting glucose, triglycerides, total cholesterol, LDL-cholesterol, hemoglobin, creatinine, potassium, total calcium, phosphorous, calcium-phosphorous product, iPTH, uric acid, Kt/V, ultrafiltration rate, and medications use including ACEI and/or ARB, Beta-blocker and calcium channel blocker.*

## Discussion

In the present study, we investigated the relationship between age and ΔHRV in diabetic HD patients. We found that pre-dialytic HF% was significantly higher in the patients ≥65 years old than in those <65 years old. In addition, the patients <65 years old had increases in VLF, LF, and HF after HD, whereas the patients ≥65 years old had an increase in LF and a decrease in HF% after HD. Diastolic blood pressure, cerebrovascular disease, fasting glucose, hemoglobin, beta-blocker use and ultrafiltration rate were significantly associated with ΔHRV in the patients <65 years old; whereas cerebrovascular disease, systolic blood pressure, fasting glucose, total cholesterol and hemoglobin were associated with ΔHRV in the patients ≥65 years old.

An increasing number of studies have investigated the effects of HD on the elderly (Anand et al., 2010). The first major finding of the current study is that VLF, LF, and HF increased significantly after HD in the patients aged <65 years, whereas LF increased during HD but HF% decreased significantly in the patients ≥65 years old. HF has been reported to represent respiratory sinus arrhythmia and vagal control of heart rate (Fouad et al., 1984). The function of autonomic nerve system of the elderly population might decline (Catai et al., 2014). Tan et al. (2017) reported a decrease in parasympathetic nervous activity during the nighttime postoperatively in elderly patients, and concluded that this may be due to higher sympathetic nervous activity resulting in a decrease in parasympathetic nervous activity. In addition, Genovesi et al. (2007) reported an increase in LF and a decrease in HF after HD, which is consistent with our findings and suggests impaired changes in the compensatory baroreflex-mediated activation of the sympathetic nervous system in elderly patients. Compared to pre-dialytic HRV, the changes of HRV during HD have been recognized as a stronger predictor of death, and impaired increase in HRV after HD was associated with mortality in HD patients (Chen et al., 2016). In our study, HRV increased significantly after HD in the patients aged <65 years, but HF% was drastically decreased after HD in patients aged ≥65 years. This finding implies that impaired parasympathetic response to HD may be partly responsible for unfavorable outcomes in the elderly HD patients. Therefore, the present study appears that patients ≥65 years old with DM had worse autonomic nervous system function after HD than patients <65 years old.

The current study also revealed that only pre-dialytic HF% was significantly higher in the patients ≥65 years old. Although HRV might be reduced with age, however, some studies showed the lack of negative correlation between age and HRV. Kalopita et al. (2014) found that HRV was not negatively correlated with age in diabetic patients. Furthermore, Amra et al. (2017) showed no correlation between age and HRV in diabetic group among patients with sleep apnea syndrome. McKune et al. (2017) even found that HF significantly increased with age in elderly people aged between 60 and 86 years, these findings are similar to the work by Reardon and Malik (1996) who were the first to examine HRV in the very elderly (age range 40–102 years). They demonstrated that while global 24-h HRV decreased with increasing age, HRV measures caused by parasympathetic modulation, such as pre-dialytic HF or HF%, did not decline with age. The real cause remains to be studied in diabetic maintenance HD patients.

In the present study, ultrafiltration rate was associated with changes in LF after HD in the patients <65 years old. The process of ultrafiltration during HD reduces circulating volume and arterial pressure, which can be detected by baroreceptors to trigger a cardiac sympathetic response. Barnas et al. (1999) investigated autonomic nervous system changes during HD, and reported that the LF component of HRV increased during non-hypotensive dialysis. Furthermore, systolic and diastolic blood pressures were associated with ΔHRV in our diabetic patients undergoing HD. Dysregulation of the autonomic nervous system can lead to sustained hypertension, and subsequently to an increased risk of vascular complications (Palatini et al., 2006; Licht et al., 2013). Hypertension has also been associated with lower HRV (Schroeder et al., 2003), which suggests that compromised sympathetic-vagal balance in patients with hypertension. In addition, fluid status may also play a role in the association between blood pressure and changes in HRV. Ferrario et al. (2015) evaluated the effect of central volume on changes in the LF parameter of HRV in patients undergoing HD, and found that patients with a low hydration status before HD had an increased LF after HD, whereas those with a high hydration status before HD exhibited no significant change in LF. A lower central volume caused by dialysis can lead to alterations in the capacity of the CV system and thereby changes in LF In patients with a low hydration status. However, removal of the same volume of fluid during HD may not reduce the central volume sufficiently to reactivate oscillations in frequency in patients with a high hydration status, and this may explain the associations between blood pressure, ultrafiltration rate and ΔHRV in this study.

An increasing number of studies have reported an association between an imbalance of the autonomic nervous system and pathophysiological conditions, including, dyslipidemia, sudden death, coronary artery disease, heart failure, and CV risk factors. In the present study, total cholesterol was associated with reduction in LF after HD in the patients aged ≥65 years old. A low HRV has been reported to able to predict intima media thickness (Dekker et al., 2000) and progression of coronary atherosclerosis (Huikuri et al., 1999) in patients with recent CV disorders. Manfrini et al. (2008) investigated whether hypercholesterolemic atherosclerosis leads to impairment of the vagus nerve and worsening of HRV (Manfrini et al., 2008), and found that an increase in plaque size and substantial arterial remodeling led to outward stretching of the vessel wall behind the plaque, and was associated with autonomic dysfunction due to impaired vagal tone. This may explain the positive association between total cholesterol and reduction in HRV after HD in patients with diabetes.

Hemoglobin level was positively correlated with change in LF after HD in patients ≥65 years old in the present study. In line with our findings, a positive correlation between hemoglobin and LF was shown in HD patients (Ng et al., 2017). This implied that better sympathetic compensation occur as a result of fluid removal during HD in patients with better hemoglobin level. Anemia might be related higher non-transferrin-bound iron and labile plasma iron levels, leading to oxidative stress-mediated cardiac autonomic dysfunction, thus resulting in reduced HRV (Wijarnpreecha et al., 2015).

A lower HRV has been reported in patients with hemorrhagic and ischemic stroke (Korpelainen et al., 1999; Colivicchi et al., 2004), and baroreflex impairment has also been reported in these patients (Sykora et al., 2008). In the current study, a history of cerebrovascular disease was associated with both decrease in VLF and HF after HD in the patients aged <65 years old, and decrease in HF% after HD in the patients aged ≥65 years old. In other words, cerebrovascular disease was associated with a smaller degree in the decrease in HF% after HD. This implies that autonomic CV dysregulation and impaired parasympathetic reaction occur as a result of fluid removal during HD in patients with stroke.

Another finding of the current study is that fasting glucose was associated with a change in HRV in the diabetic patients. Zitt et al. (2008) investigated the effect of DM on blunted autonomic response in patients undergoing HD, this suggests that impaired autonomic function may be associated with damage caused by diabetes in autonomic neuropathy. In the current study, we also found an association between a high level of fasting glucose and a smaller degree of change in HF% after HD, implying that patients with diabetes and a high level of fasting glucose may have an impaired sympathy-vagal equilibrium. Microangiopathy has also been reported to play an important role in the development of diabetic neuropathy (Wada et al., 1999, 2001). The advanced glycation end products interacts with their receptors, can have a biological effect on tissues resulting in the complications of diabetes, further contributing to endoneural vascular dysfunction, consequently peripheral nerve microangiopathy (Wada and Yagihashi, 2005) and peripheral neuropathy (Singh et al., 2014).

There are several limitations to the present study. First, HRV can be measured in frequency or time domain parameters; however, we only used the frequency domain HRV. Although frequency domain parameters have been reported to be well correlated (*r* = 0.85) with time domain parameters, several time domain parameters have been reported to be clinically relevant (Kleiger et al., 1987, 1991). Thus, time domain measurements need to be included in future studies. Second, spectral HRV measurements and their physiological interpretation are not clear as previously thought, and they also have many limitations. More recent measures of HRV (e.g., fractal/complexity/scale invariant measures, entropy-based measures) should also be investigated as spectral HRV measurements in future studies. Third, causal relationships in this cross-sectional study could not be confirmed, and future prospective studies are needed to address this issue. Fourth, the number of enrolled patients was relatively small, which may have limited the statistical power. The percentage of non-stationary segments was lacking. Finally, the circadian pattern of autonomic modulation of the heart rate has been reported to exhibit a reduced HRV during the day with increased sympathetic activity and increased HRV during the night with the predominance of vagal modulation (Molgaard et al., 1991; Chen et al., 2011). In the present study, in order to minimize the influence of the circadian rhythm, we performed all HRV examinations during the day (between 8 a.m. and 5 p.m.), however, the rhythm may have been lost in some patients and the influence could not be defined. A longer period of ECG recordings may have established a baseline for each patient.

## Conclusion

Our results demonstrated significant changes in HRV after HD in diabetic patients. In the patients aged ≥65 years, LF was increased, whereas HF% was decreased significantly after HD. Among the HRV parameters, age had an interaction with the change of HF%, suggesting impaired parasympathetic response to HD in the elderly patients. Moreover, systolic blood pressure, total cholesterol, hemoglobin, and hemoglobin were associated with ΔLF, whereas cerebrovascular disease, systolic blood pressure, and fasting glucose were associated with ΔHF% in patients aged ≥65 years. Further prospective studies and follow-up are needed to validate the effects of aging and HRV on the outcomes of patients with diabetes.

## Author Contributions

Conceived and designed the experiments and analyzed the data: S-WN, J-CH, and S-CC. Performed the experiments: S-WN, J-CH, HL, and I-CK. Contributed reagents/materials/analysis tools: J-CH, P-YW, S-CC, Y-WC, and J-MC. Wrote the manuscript: S-WN and J-CH.

## Conflict of Interest Statement

The authors declare that the research was conducted in the absence of any commercial or financial relationships that could be construed as a potential conflict of interest.
